# Direct Insertion of the Papillary Muscle into the Anterior Mitral Leaflet: Cadaveric Findings

**DOI:** 10.7759/cureus.1752

**Published:** 2017-10-05

**Authors:** Sarvenaz Sheikh, Joe Iwanaga, Jocelyn R Gonzales, Tsuyoshi Saga, Marios Loukas, Rod J Oskouian, R. Shane Tubbs

**Affiliations:** 1 Seattle Science Foundation; 2 Neurosurgery, Seattle Science Foundation; 3 Department of Anatomy, Kurume University School of Medicine; 4 Department of Anatomical Sciences, St. George's University School of Medicine, Grenada, West Indies; 5 Neurosurgery, Complex Spine, Swedish Neuroscience Institute

**Keywords:** mitral valve, direct insertion of the papillary muscle into the mitral valve, papillary muscle, malfunctions of cardiac anatomy, dpm

## Abstract

Direct insertion of the anterior papillary muscle (DPM) into the mitral valve is uncommon. During the routine dissection of an adult female, a DPM into the mitral valve with abnormal chordae tendinae was observed. This abnormal papillary muscle was measured as 28.0 mm in length from myocardial to insertion, 14.8 mm in width, and 7.0 mm in depth. The embryology, symptoms, associated cardiac diseases, and surgical precautions of this congenital malformation in the heart are reviewed.

## Introduction

The chordae tendinae of the mitral valve project from the papillary muscles, connect to the leaflets, and impact the stress state and shape of the valve [1]. Many cardiac diseases are associated with disorders of the mitral valve and include prolapse and stenosis [2]. We report cadaveric findings of the direct insertion of the papillary muscle (DPM) into the anterior mitral valve leaflet found during routine anatomy dissection. 

## Case presentation

**Figure 1:**
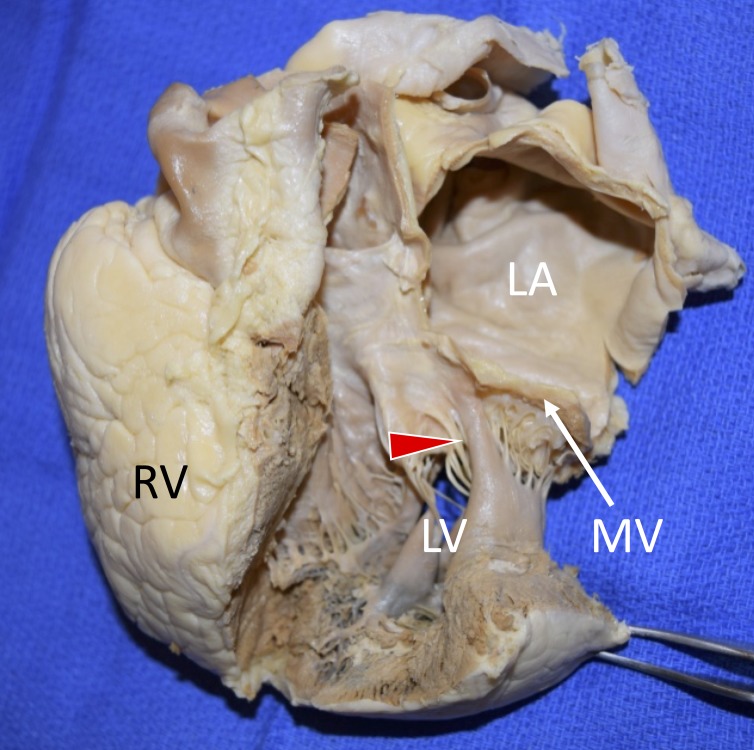
Anterior view of the left atrium and ventricle. Note that papillary muscle directly inserts into anterior leaflet of the mitral valve (arrowhead). LA; left atrium, LV; left ventricle, MV; mitral valve, RV; right ventricle

During routine dissection of the heart, a DPM of the anterior papillary muscle into the mitral valve was observed in the heart of an elderly female (Figure 1).

**Figure 2:**
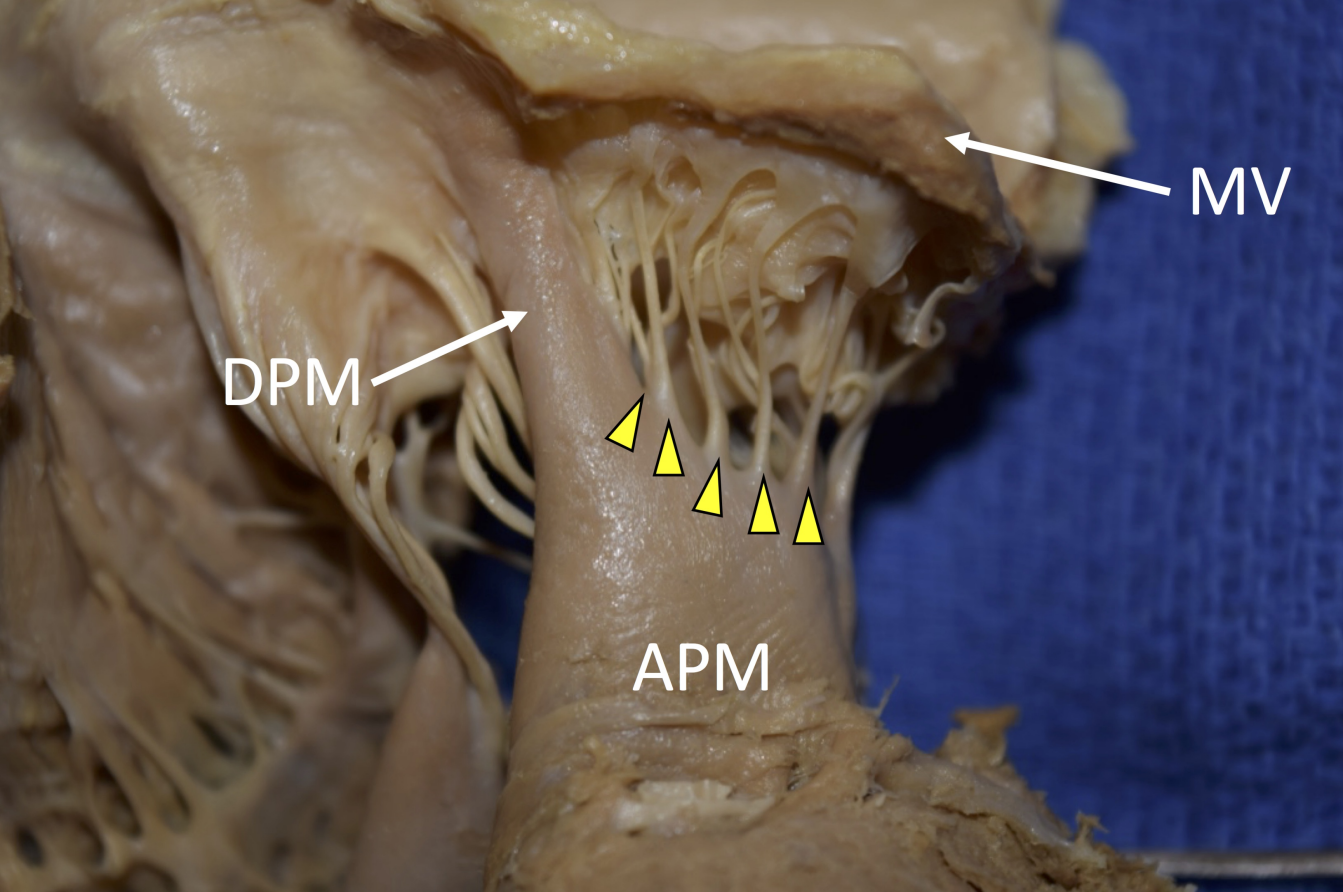
Direct insertion of the anterior papillary muscle (DPM) and normal chordae tendinae (arrowheads). Note that normal five chordae tendinae insert into anterior leaflet of the mitral valve. APM; anterior papillary muscle, MV; mitral valve

The patient had died of natural causes and there were no signs of dilation of any of the heart chambers. There was an age-appropriate degree of coronary arteriosclerosis but no signs of cardiac ischemia. This abnormal papillary muscle was found to have a length of 28.0 mm from the myocardial to valve insertion, and was 14.8 mm in width, and 7.0 mm in depth. The extension itself was 14.3 mm in length and 5.2 mm in width. The size of the heart was normal and five short chordae tendinae were extended from this abnormal anterior papillary muscle (Figure 2).

**Figure 3:**
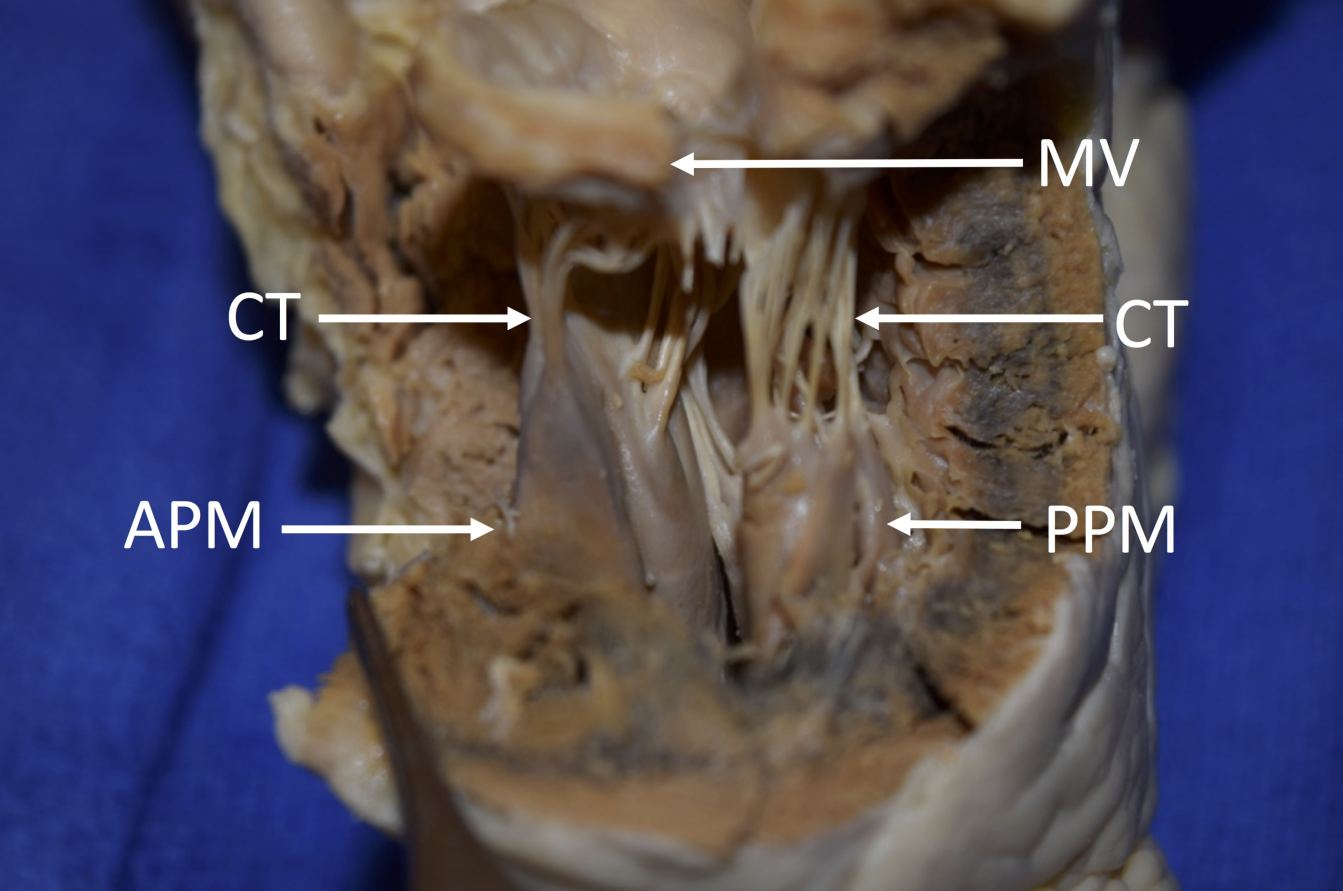
Lateral view of the left ventricle. APM; anterior papillary muscle, CT; chordae tendinae, MV; mitral valve, PPM; posterior papillary muscle

The posterior papillary muscles of the left ventricle were measured as 7.5 mm and 7.6 mm in width, and 15.7 mm and 18.6 mm in length (Figure 3).

The mitral valve was not tethered by the DPM while positioning the valve into a normal, closed position. No other congenital anomalies were found in this specimen.

## Discussion

In our case, a DPM was identified in a cadaveric specimen and this finding affords the opportunity to examine such an anatomical variant in more detail. A DPM has a reported prevalence of 0.35-13.00% and has also been called the mitral arcade [3-7].

In developing hearts, there is a semi-circle shaped myocardial ridge in which the anterior and posterior parts become the papillary muscles. During week eight of embryological development, the ridge begins to delaminate and transform into the papillary muscles. In week 10, within cushion tissue of the developing mural and aortic leaflets, there is a small gap between where the cushion tissue connects to the tips of the developing papillary muscle. During weeks 10-13, the chordae tendinae are formed. It is during this time that if there are no chordae tendinae or the tendinae are abnormally short, then the papillary muscle will fuse with the mitral valve directly [1].

Most cases of a DPM into the mitral valve have been reported to have concomitant hemodynamic disturbances [8]. Associated pathologies include myocardial hypertrophy, enlarged hearts, asymmetric thickening of the septum, and most commonly, left ventricular outflow obstruction [2-3]. However, in the present case, we observed a normal sized heart and only slightly enlarged left anterior papillary muscle (Figure 2). According to Ozbag, et al. [9], the left anterior papillary muscle has a mean of 33.6 mm in length and 15.2 mm in width. In a study of a Bangladeshi population, 80 cadavers were examined and the left anterior papillary muscles were found to have a mean length of 18.9 mm [10]. Roberts, et al. [6], reported (from 12 normal hearts) an average of 12 chordae tendinae per each left ventricular papillary muscle. In comparison, our specimen had five tendinae connected to the abnormal left anterior papillary muscle. Some have found that such an anatomical derailment leads to the inflexibility of the papillary muscles and mitral leaflets, and increases the left ventricular outflow tract pressure [6].

Symptoms of a patient with this mitral valve anomaly include precordial systolic ejection murmur, the hypostasis of posterior parts of the body, breathlessness, fatigue, and effects similar to valvular heart disease [4, 8]. The anomalous papillary muscle directly inserted into the mitral valve could result in obstruction to the left ventricular outflow, an absence of mitral systolic anterior motion, contact of a portion of the anterior leaflet with the septum, and mitral regurgitation [2].

## Conclusions

We report a DPM attaching into the anterior mitral valve leaflet found during routine anatomy dissection. A cadaveric observation of a DPM offers an interesting window into more precisely examining these uncommon congenital findings. Additionally, as this finding has been linked to hemodynamic issues resulting in myocardial hypertrophy, heart enlargement, thickened interventricular septum, inflexibility of the papillary muscle with resultant increases in left ventricular outflow pressures, mitral valve regurgitation, and outflow obstruction of the left ventricle, identifying them on imaging might warrant closer follow-up of patients. Further anatomical and clinical studies that evaluate the anatomy and functional consequences of a DPM attachment into the anterior leaflet of the mitral valve are now necessary.
